# 1,5-Bis(1-phenyl­ethyl­idene)carbonohydrazide

**DOI:** 10.1107/S1600536810042121

**Published:** 2010-10-23

**Authors:** Lingqian Kong, Yan Qiao, Zhiqing Gao, Xiuping Ju

**Affiliations:** a Dongchang College, Liaocheng University, Liaocheng 250059, People’s Republic of China

## Abstract

In the title mol­ecule, C_17_H_18_N_4_O, the two phenyl rings form a dihedral angle of 18.15 (17)°. In the crystal, pairs of inter­molecular N—H⋯O hydrogen bonds link the mol­ecules into centrosymmetric dimers. Weak inter­molecular C—H⋯O inter­actions further link the dimers into chains running along [010].

## Related literature

For related structures, see: Qiao *et al.* (2010 ▶); Kolb *et al.* (1994 ▶4); Meyers *et al.* (1995 ▶).
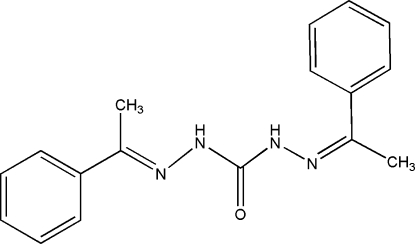


## Experimental

### 

#### Crystal data

C_17_H_18_N_4_O
*M* *_r_* = 294.35
Monoclinic, 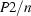

*a* = 12.9393 (12) Å
*b* = 5.4858 (5) Å
*c* = 22.703 (2) Å
β = 104.681 (1)°
*V* = 1558.9 (2) Å^3^
*Z* = 4
Mo *K*α radiation
μ = 0.08 mm^−1^
*T* = 298 K
0.50 × 0.31 × 0.25 mm

#### Data collection

Bruker SMART APEX CCD area-detector diffractometer
Absorption correction: multi-scan (*SADABS*; Sheldrick, 1996 ▶) *T*_min_ = 0.960, *T*_max_ = 0.980
7406 measured reflections
2757 independent reflections
1389 reflections with *I* > 2σ(*I*)
*R*_int_ = 0.048

#### Refinement

*R*[*F* ^2^ > 2σ(*F* ^2^)] = 0.049
*wR*(*F* ^2^) = 0.148
*S* = 0.90
2757 reflections
201 parameters
1 restraint
H-atom parameters constrained
Δρ_max_ = 0.18 e Å^−3^
Δρ_min_ = −0.13 e Å^−3^

### 

Data collection: *SMART* (Bruker, 2007 ▶); cell refinement: *SAINT* (Bruker, 2007 ▶); data reduction: *SAINT* program(s) used to solve structure: *SHELXS97* (Sheldrick, 2008 ▶); program(s) used to refine structure: *SHELXL97* (Sheldrick, 2008 ▶); molecular graphics: *SHELXTL* (Sheldrick, 2008 ▶); software used to prepare material for publication: *SHELXTL*.

## Supplementary Material

Crystal structure: contains datablocks I, global. DOI: 10.1107/S1600536810042121/cv2776sup1.cif

Structure factors: contains datablocks I. DOI: 10.1107/S1600536810042121/cv2776Isup2.hkl

Additional supplementary materials:  crystallographic information; 3D view; checkCIF report

## Figures and Tables

**Table 1:** Hydrogen-bond geometry (Å, °)

*D*—H⋯*A*	*D*—H	H⋯*A*	*D*⋯*A*	*D*—H⋯*A*
C3—H3*C*⋯O5^i^	0.96	2.53	3.405 (3)	151
N2—H2⋯O5^ii^	0.86	2.11	2.955 (3)	166

Symmetry codes: (i) 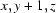; (ii) 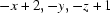.

**Fig. 1.**
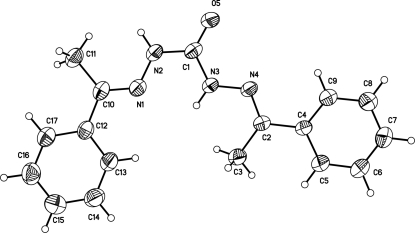
A view of (I) showing the atomic numbering scheme and 30% probability displacement ellipsoids.

## supplementary crystallographic information

### Comment

In continuation of our study of Schiff bases and carbonohydrazides (Qiao *et al.*, 2010), we obtained the title compound, (I), and present here its crystal structure.

In (I) (Fig. 1), the bond lengths and angles are normal and comparable to those observed in  bis(4-methoxyphenylmethine)carbonohydrazide (Kolb *et al.*, 1994) and bis(3-fluorophenylmethine)carbonohydrazide (Meyers *et al.*, 1995). The C=N bond lengths are 1.282 (3) ° and 1.286 (3)° (C10=N1 and C2=N4, respectively) showing their double-bond character. Two phenyl rings - C4-C9 and C12—C17, respectively - form a dihedral angle of 18.15 (17)°.

In the crystal structure, intermolecular N—H···O hydrogen bonds (Table 1) link the molecules into centrosymmetric dimers, and weak intermolecular C—H···O interactions (Table 1) link further these dimers into chains running in direction [010].

### Experimental

Acetophenone (10.0 mmol) and carbohydrazide (5.0 mmol) were mixed in 50 ml flash under sovlent-free condtions. After stirring for 3 h at 373 K, the resulting mixture was cooled to room temperature, and recrystalized from ethanol, and afforded the title compound as a crystalline solid.

### Refinement

All H atoms were placed in geometrically idealized positions (N—H = 0.86 and C—H = 0.93–0.96 Å) and treated as riding on their parent atoms, with *U*_iso_(H) = 1.2–1.5*U*_eq_(C, N).

### Crystal data

**Table:**

C_17_H_18_N_4_O	*F*(000) = 624
*M**_r_* = 294.35	*D*_x_ = 1.254 Mg m^−^^3^
Monoclinic, *P*2/*n*	Mo *K*α radiation, λ = 0.71073 Å
*a* = 12.9393 (12) Å	Cell parameters from 1233 reflections
*b* = 5.4858 (5) Å	θ = 2.9–21.2°
*c* = 22.703 (2) Å	µ = 0.08 mm^−^^1^
β = 104.681 (1)°	*T* = 298 K
*V* = 1558.9 (2) Å^3^	Block, colourless
*Z* = 4	0.50 × 0.31 × 0.25 mm

### Data collection

**Table:**

Bruker SMART APEX CCD area-detector diffractometer	2757 independent reflections
Radiation source: fine-focus sealed tube	1389 reflections with *I* > 2σ(*I*)
graphite	*R*_int_ = 0.048
phi and ω scans	θ_max_ = 25.0°, θ_min_ = 2.9°
Absorption correction: multi-scan (*SADABS*; Sheldrick, 1996)	*h* = −15→15
*T*_min_ = 0.960, *T*_max_ = 0.980	*k* = −6→6
7406 measured reflections	*l* = −26→16

### Refinement

**Table:**

Refinement on *F*^2^	Primary atom site location: structure-invariant direct methods
Least-squares matrix: full	Secondary atom site location: difference Fourier map
*R*[*F*^2^ > 2σ(*F*^2^)] = 0.049	Hydrogen site location: inferred from neighbouring sites
*wR*(*F*^2^) = 0.148	H-atom parameters constrained
*S* = 0.90	*w* = 1/[σ^2^(*F*_o_^2^) + (0.0747*P*)^2^] where *P* = (*F*_o_^2^ + 2*F*_c_^2^)/3
2757 reflections	(Δ/σ)_max_ < 0.001
201 parameters	Δρ_max_ = 0.18 e Å^−^^3^
1 restraint	Δρ_min_ = −0.13 e Å^−^^3^

### Special details

**Table:**

Geometry. All e.s.d.'s (except the e.s.d. in the dihedral angle between two l.s. planes) are estimated using the full covariance matrix. The cell e.s.d.'s are taken into account individually in the estimation of e.s.d.'s in distances, angles and torsion angles; correlations between e.s.d.'s in cell parameters are only used when they are defined by crystal symmetry. An approximate (isotropic) treatment of cell e.s.d.'s is used for estimating e.s.d.'s involving l.s. planes.
Refinement. Refinement of *F*^2^ against ALL reflections. The weighted *R*-factor *wR* and goodness of fit *S* are based on *F*^2^, conventional *R*-factors *R* are based on *F*, with *F* set to zero for negative *F*^2^. The threshold expression of *F*^2^ > σ(*F*^2^) is used only for calculating *R*-factors(gt) *etc*. and is not relevant to the choice of reflections for refinement. *R*-factors based on *F*^2^ are statistically about twice as large as those based on *F*, and *R*- factors based on ALL data will be even larger.

### Fractional atomic coordinates and isotropic or equivalent isotropic displacement parameters (Å^2^)

**Table:**

	*x*	*y*	*z*	*U*_iso_*/*U*_eq_	
O5	0.86754 (12)	0.0739 (3)	0.46156 (9)	0.0764 (6)	
N1	0.96224 (16)	0.4666 (4)	0.58368 (10)	0.0631 (6)	
N2	0.96359 (15)	0.2825 (4)	0.54321 (10)	0.0679 (6)	
H2	1.0196	0.1938	0.5466	0.082*	
N3	0.79610 (14)	0.4095 (4)	0.49611 (9)	0.0668 (6)	
H3	0.8087	0.5299	0.5212	0.080*	
N4	0.69764 (15)	0.3902 (4)	0.45574 (9)	0.0596 (6)	
C1	0.87369 (18)	0.2428 (5)	0.49730 (13)	0.0614 (7)	
C2	0.63193 (18)	0.5649 (4)	0.45685 (10)	0.0542 (6)	
C3	0.65842 (18)	0.7846 (5)	0.49701 (13)	0.0795 (8)	
H3A	0.6503	0.7461	0.5368	0.119*	
H3B	0.6111	0.9157	0.4800	0.119*	
H3C	0.7309	0.8329	0.5000	0.119*	
C4	0.52443 (18)	0.5423 (5)	0.41536 (11)	0.0551 (6)	
C5	0.4445 (2)	0.7041 (6)	0.41690 (14)	0.0928 (10)	
H5	0.4592	0.8337	0.4442	0.111*	
C6	0.3434 (2)	0.6811 (7)	0.37943 (17)	0.1109 (12)	
H6	0.2909	0.7936	0.3818	0.133*	
C7	0.3200 (2)	0.4948 (7)	0.33899 (14)	0.0934 (10)	
H7	0.2518	0.4792	0.3133	0.112*	
C8	0.3977 (2)	0.3314 (6)	0.33648 (14)	0.1002 (11)	
H8	0.3828	0.2028	0.3089	0.120*	
C9	0.4984 (2)	0.3561 (6)	0.37467 (13)	0.0836 (9)	
H9	0.5503	0.2416	0.3726	0.100*	
C10	1.04459 (19)	0.5073 (5)	0.62766 (12)	0.0618 (7)	
C11	1.14821 (18)	0.3686 (5)	0.63824 (12)	0.0783 (8)	
H11A	1.1447	0.2260	0.6622	0.117*	
H11B	1.2060	0.4706	0.6595	0.117*	
H11C	1.1600	0.3207	0.5998	0.117*	
C12	1.03396 (18)	0.7066 (5)	0.66970 (11)	0.0613 (7)	
C13	0.9504 (2)	0.8704 (6)	0.65627 (13)	0.0774 (8)	
H13	0.8986	0.8551	0.6197	0.093*	
C14	0.9416 (2)	1.0546 (6)	0.69527 (16)	0.0880 (9)	
H14	0.8835	1.1601	0.6853	0.106*	
C15	1.0179 (3)	1.0851 (6)	0.74911 (15)	0.0845 (9)	
H15	1.0131	1.2126	0.7753	0.101*	
C16	1.1002 (2)	0.9256 (7)	0.76324 (14)	0.0906 (10)	
H16	1.1521	0.9426	0.7998	0.109*	
C17	1.1083 (2)	0.7385 (6)	0.72431 (14)	0.0815 (9)	
H17	1.1655	0.6309	0.7352	0.098*	

### Atomic displacement parameters (Å^2^)

**Table:**

	*U*^11^	*U*^22^	*U*^33^	*U*^12^	*U*^13^	*U*^23^
O5	0.0605 (11)	0.0704 (14)	0.0975 (14)	0.0118 (9)	0.0187 (10)	−0.0167 (11)
N1	0.0542 (12)	0.0619 (15)	0.0726 (14)	0.0052 (11)	0.0148 (11)	0.0028 (12)
N2	0.0497 (12)	0.0686 (16)	0.0815 (15)	0.0129 (11)	0.0094 (12)	0.0009 (13)
N3	0.0482 (12)	0.0621 (15)	0.0854 (15)	0.0119 (11)	0.0083 (11)	−0.0128 (12)
N4	0.0495 (11)	0.0583 (14)	0.0715 (13)	0.0102 (10)	0.0163 (10)	−0.0017 (11)
C1	0.0501 (15)	0.0579 (19)	0.0787 (18)	0.0091 (14)	0.0209 (14)	0.0069 (15)
C2	0.0537 (14)	0.0501 (17)	0.0622 (15)	0.0050 (12)	0.0208 (13)	0.0021 (13)
C3	0.0647 (16)	0.064 (2)	0.105 (2)	0.0061 (14)	0.0140 (15)	−0.0156 (17)
C4	0.0546 (14)	0.0531 (17)	0.0595 (15)	0.0097 (12)	0.0178 (12)	0.0005 (13)
C5	0.0669 (18)	0.080 (2)	0.120 (2)	0.0188 (16)	0.0031 (18)	−0.0300 (19)
C6	0.069 (2)	0.109 (3)	0.139 (3)	0.0329 (19)	−0.003 (2)	−0.027 (3)
C7	0.0665 (18)	0.113 (3)	0.089 (2)	0.014 (2)	−0.0036 (16)	−0.011 (2)
C8	0.086 (2)	0.109 (3)	0.093 (2)	0.022 (2)	−0.0016 (19)	−0.032 (2)
C9	0.0678 (18)	0.093 (2)	0.0825 (19)	0.0223 (16)	0.0058 (16)	−0.0227 (19)
C10	0.0493 (14)	0.0676 (19)	0.0676 (17)	0.0015 (13)	0.0133 (14)	0.0164 (15)
C11	0.0550 (14)	0.091 (2)	0.0872 (19)	0.0137 (15)	0.0146 (14)	0.0071 (17)
C12	0.0480 (14)	0.0679 (19)	0.0677 (17)	−0.0023 (13)	0.0139 (13)	0.0092 (15)
C13	0.0674 (17)	0.076 (2)	0.083 (2)	0.0100 (16)	0.0071 (15)	0.0017 (18)
C14	0.079 (2)	0.081 (2)	0.104 (2)	0.0140 (17)	0.023 (2)	−0.001 (2)
C15	0.083 (2)	0.082 (2)	0.094 (2)	−0.0121 (18)	0.0339 (19)	−0.0083 (19)
C16	0.0691 (19)	0.116 (3)	0.083 (2)	−0.004 (2)	0.0131 (17)	−0.006 (2)
C17	0.0582 (16)	0.095 (2)	0.087 (2)	0.0092 (16)	0.0111 (16)	−0.0002 (19)

### Geometric parameters (Å, °)

**Table:**

O5—C1	1.221 (3)	C7—H7	0.9300
N1—C10	1.282 (3)	C8—C9	1.377 (3)
N1—N2	1.368 (3)	C8—H8	0.9300
N2—C1	1.368 (3)	C9—H9	0.9300
N2—H2	0.8600	C10—C12	1.480 (4)
N3—C1	1.353 (3)	C10—C11	1.507 (3)
N3—N4	1.372 (2)	C11—H11A	0.9600
N3—H3	0.8600	C11—H11B	0.9600
N4—C2	1.286 (3)	C11—H11C	0.9600
C2—C4	1.474 (3)	C12—C17	1.374 (3)
C2—C3	1.498 (3)	C12—C13	1.379 (3)
C3—H3A	0.9600	C13—C14	1.367 (4)
C3—H3B	0.9600	C13—H13	0.9300
C3—H3C	0.9600	C14—C15	1.373 (4)
C4—C9	1.361 (3)	C14—H14	0.9300
C4—C5	1.370 (3)	C15—C16	1.353 (4)
C5—C6	1.374 (4)	C15—H15	0.9300
C5—H5	0.9300	C16—C17	1.376 (4)
C6—C7	1.356 (4)	C16—H16	0.9300
C6—H6	0.9300	C17—H17	0.9300
C7—C8	1.358 (4)		
			
C10—N1—N2	120.1 (2)	C7—C8—H8	120.0
C1—N2—N1	118.4 (2)	C9—C8—H8	120.0
C1—N2—H2	120.8	C4—C9—C8	122.2 (3)
N1—N2—H2	120.8	C4—C9—H9	118.9
C1—N3—N4	121.3 (2)	C8—C9—H9	118.9
C1—N3—H3	119.3	N1—C10—C12	115.9 (2)
N4—N3—H3	119.3	N1—C10—C11	124.5 (3)
C2—N4—N3	115.9 (2)	C12—C10—C11	119.6 (2)
O5—C1—N3	125.2 (2)	C10—C11—H11A	109.5
O5—C1—N2	121.8 (2)	C10—C11—H11B	109.5
N3—C1—N2	113.0 (3)	H11A—C11—H11B	109.5
N4—C2—C4	116.5 (2)	C10—C11—H11C	109.5
N4—C2—C3	124.2 (2)	H11A—C11—H11C	109.5
C4—C2—C3	119.3 (2)	H11B—C11—H11C	109.5
C2—C3—H3A	109.5	C17—C12—C13	116.5 (3)
C2—C3—H3B	109.5	C17—C12—C10	121.2 (2)
H3A—C3—H3B	109.5	C13—C12—C10	122.3 (2)
C2—C3—H3C	109.5	C14—C13—C12	121.8 (3)
H3A—C3—H3C	109.5	C14—C13—H13	119.1
H3B—C3—H3C	109.5	C12—C13—H13	119.1
C9—C4—C5	116.4 (2)	C13—C14—C15	120.5 (3)
C9—C4—C2	121.9 (2)	C13—C14—H14	119.7
C5—C4—C2	121.7 (2)	C15—C14—H14	119.7
C4—C5—C6	122.1 (3)	C16—C15—C14	118.5 (3)
C4—C5—H5	119.0	C16—C15—H15	120.7
C6—C5—H5	119.0	C14—C15—H15	120.7
C7—C6—C5	120.2 (3)	C15—C16—C17	120.9 (3)
C7—C6—H6	119.9	C15—C16—H16	119.5
C5—C6—H6	119.9	C17—C16—H16	119.5
C6—C7—C8	119.0 (3)	C12—C17—C16	121.7 (3)
C6—C7—H7	120.5	C12—C17—H17	119.2
C8—C7—H7	120.5	C16—C17—H17	119.2
C7—C8—C9	120.1 (3)		

### Hydrogen-bond geometry (Å, °)

**Table:**

*D*—H···*A*	*D*—H	H···*A*	*D*···*A*	*D*—H···*A*
C3—H3C···O5^i^	0.96	2.53	3.405 (3)	151
N2—H2···O5^ii^	0.86	2.11	2.955 (3)	166

Symmetry codes: (i) *x*, *y*+1, *z*; (ii) −*x*+2, −*y*, −*z*+1.
